# Operational and Financial Impacts of Digital Health Technology: A Study on Canadian Healthcare System during the COVID-19 Pandemic

**DOI:** 10.3390/ijerph192215025

**Published:** 2022-11-15

**Authors:** Zixin (Jessie) Jin, Zongjie Wang

**Affiliations:** 1 Faculty of Arts and Science, Havergal College, Toronto, ON M5N 2H9, Canada; 2 Faculty of Pharmacy, University of Toronto, Toronto, ON M5S 3M2, Canada; 3 Feinberg School of Medicine, Northwestern University, Chicago, IL 60611, USA

**Keywords:** digital health, hospital costs, patient experience, virtual care

## Abstract

During COVID-19, hospital capacity was significantly reduced to limit the spread of the pandemic. The limitations affected the efficiency of service delivery. We examined the effects of pandemic-related challenges on patient experience and hypothesize that digital health implementation increased patient satisfaction. We surveyed nationally aggregated data in hospital occupancy, hospital funding and patient experience, and plotted their correlation. We found digital health to contribute to patient experience and service-delivery effectiveness. We evaluate the benefits of digital health in context of hospital service delivery. Post-COVID-19, we recommend a continued implementation of digital health and offer suggestions to further improve its efficiency and cost-effectiveness.

## 1. Introduction

The coronavirus disease 2019 (COVID-19) pandemic has had tremendous impacts on healthcare systems worldwide. The World Health Organization (WHO) recommended healthcare services to limit in-person interactions to urgent services [1]. Adaptation to COVID-19 protocols challenged hospitals to reallocate resources to areas of greatest need and to accommodate COVID-19 patients [2]. Restrictions posed by the pandemic caused limitations to healthcare services and created a significant financial burden for health systems. Pandemic-related challenges affected the quality of hospital service deliveries and, in turn, impacted patient experiences.

Research to date has examined the effect of COVID-19 on healthcare services across jurisdictions, revealing COVID-19′s impact on various functions of healthcare services such as hospitals, healthcare provision and drug administrations. Globally, the World Health Organization revealed healthcare adoption priorities around clinical health systems in reaction to the pandemic [3]. In the United Kingdom, major studies have highlighted healthcare delivery and clinical management as priorities in COVID-19 healthcare system response and marked a shift in health financing to include digital technology [4,5,6]. In the United States, COVID-19 response objectives include resource reallocations, access and guidelines for telehealth, and data integration on digital health platforms, according to the National Institute of Health [7]. US-focused studies have indicated digital health integrations as an opportunity to enhance the delivery of health services [8,9,10]. The research to date highlights changes in the healthcare system from COVID-19, with digital integration being a common focus amongst relevant international studies. 

The Canadian healthcare system also largely conforms to COVID-19 prioritization in the jurisdictions highlighted above, implementing similar mechanisms in adaptation to pandemic-related constraints. The Canadian Institutes of Health Research’s Institute of Health Services and Policy Research (IHSPR) conducted a series of analyses of health-policy development, identifying virtual care as one of the seven COVID-19 priorities [11]. Their findings, alongside other studies, make clear the significance of virtual care in providing healthcare service access and meeting patient needs without the risk of disease transmission [11,12]. Such findings highlight the need for further research on the type and extent of virtual healthcare services across provinces, as well as the access, cost, and consequences in expanded use of virtual care models.

Although the research to date has highlighted the significant potential of digital healthcare, the lack of discussion on specific steps towards implementation hinders the ability to examine the practicality of implementation. Moreover, the lack of data analysis specific to the Canadian healthcare data limits the scope of discussion in Canadian implementation of digital healthcare. In this paper, we examine the effects of funding changes and pandemic restrictions on patient experience. We hypothesize digital health’s contribution to patient experience and advocate for broader implementations in the delivery of health services.

## 2. Materials and Methods

### 2.1. Source Data

We acquired the raw data for analysis from the following governmental resources:

- Canadian Institute for Health Information

https://www.cihi.ca/sites/default/files/document/series-d1-2021-en.xlsx, accessed on 23 August 2022.

https://www.cihi.ca/en/patient-experience/reporting-patient-experience-data, accessed on 21 August 2022.

https://www.cihi.ca/sites/default/files/document/impact-covid-19-on-hospital-services-march-2020-to-june-2021-data-tables-en.xlsx, accessed on 23 August 2022.

- Financial Accountability Office of Ontario

https://www.fao-on.org/en/Blog/Publications, accessed on 17 August 2022.

http://www.fao-on.org/en/Blog/Publications/health-2020, accessed on 17 August 2022.

- Government of Alberta

https://www.alberta.ca/budget-highlights.aspx, accessed on 14 August 2022.

- Government of British Columbia

https://bcbudget.gov.bc.ca/, accessed on 14 August 2022.

- Government of Canada

https://health-infobase.canada.ca/covid-19/, accessed on 11 August 2022.

- Government of Manitoba

https://www.gov.mb.ca/sharethehealth/index.html, accessed on 10 August 2022.

- Government of New Brunswick

https://www2.gnb.ca/content/gnb/en/departments/finance/budget.html, accessed on 10 August 2022.

- Government of Nova Scotia

https://novascotia.ca/news/search/?deptnum=148&month=&year=2022&q=&condition=AND, accessed on 10 August 2022.

### 2.2. Workflow

**Figure 1:**
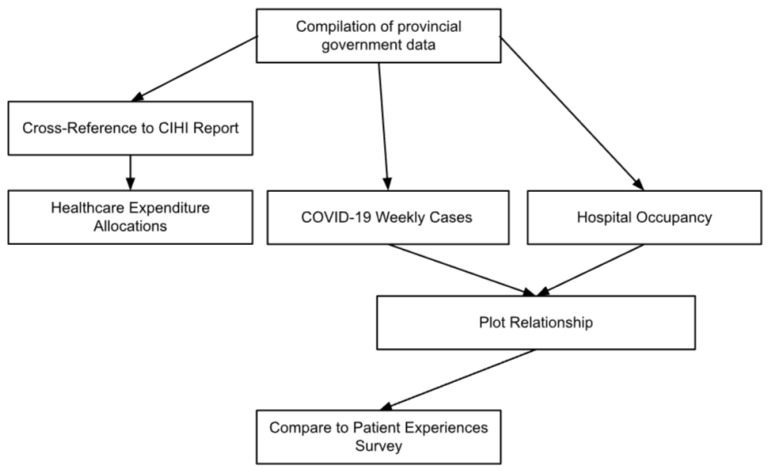
Workflow of data acquisition, analysis and comparison.

The general workflow of this work is illustrated in Figure 1. In brief, we compiled data from provincial government reports and found key parameters of health expenditure, hospital occupancy, COVID-19 cases, and patient experience. We compiled healthcare expenditure data from provincial governments and cross-referenced them with the Canadian Institute for Health Information allocation percentage report for provincial and national level healthcare expenditure allocations from 2017 to 2022 (2020–2021 and 2021–2022 data are official estimates). We plotted the funding allocation with the years to observe provincial funding trends. We also obtained data on COVID-19 weekly case confirmations from March 2020 to July 2021 from the Government of Canada. We plotted this data with the provincial level hospital occupancy, compiled from provincial government reports, to observe changes in hospital occupancy during pandemic waves. Additionally, we used the Canadian Patient Experiences Survey from 2019 to 2021, collected by the CIHI. The information is based on patient-reported experience measures (PREMs), and provincial-level responses have been adjusted to account for sample size differences and other variations.

### 2.3. Software

Our data analysis was conducted with Microsoft Excel (version 16.44) and its built-in tools (Redmond, WA, USA). Microsoft PowerPoint (version number 16) (Redmond, WA, USA), GraphPad Prism (version 9.4.1) (San Diego, CA, USA), and BioRender (version 2022) (Toronto, ON, Canada) were used to display results.

## 3. Results

### Hospital Service and Patient Experience under COVID-19

**Figure 2:**
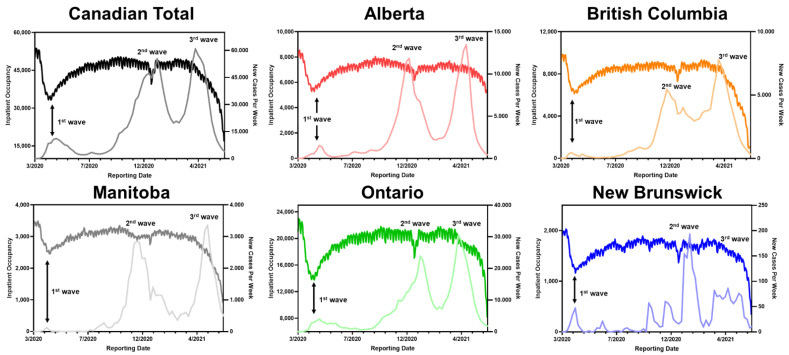
Hospital inpatient occupancy and confirmed COVID-19 cases per week, showing a correlation. Arrows indicate “COVID-19 waves”, the periods of surges in confirmed COVID-19 cases. Left axis: hospital occupancy. Right axis: confirmed new cases.

Due to the reduction in hospital capacity under COVID-19 conditions, the hospital system was forced to adapt to prioritizing lifesaving treatments, shifting most of human resources to support areas of greatest need [2]. In the pandemic period, hospital visits dropped by 9300 per day on average and 560,000 fewer surgeries were performed in Canada [13]. A significant number of surgeries were cancelled or delayed, with overall surgery levels falling by 47% compared with pre-pandemic levels [13]. Figure 2 illustrates large decline in hospital occupancy during COVID-19 waves. We found that hospital inpatient occupancy and confirmed COVID case averages were inversely correlated, with hospital occupancy levels dropping in pandemic waves. Most of the provinces demonstrated the largest inpatient occupancy drop in the first wave of the pandemic. The relationship between inpatient occupancy and confirmed COVID-19 cases per week were analyzed in numerous studies, suggesting a surged demand for, and restrained access to, hospital occupancy [14,15,16]; Increased COVID-19 cases increased the need for hospitalization and the need for inpatient occupancy limits within hospitals. As a result, hospital visits and occupancies were limited, lengthening the time before patients received treatment. 

**Figure 3:**
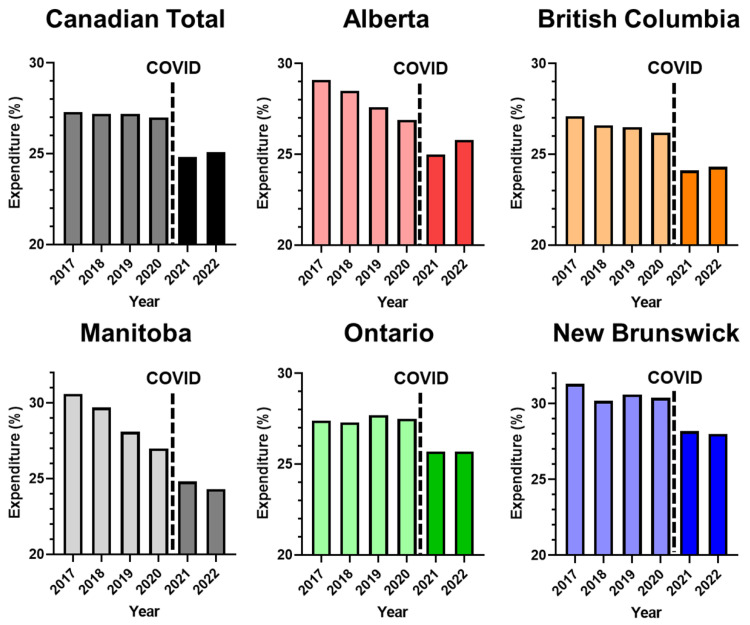
Percentage of healthcare expenditure allocated towards hospital spending, from 2017–2022. Data for 2021–2022 are official estimates.

Financially, COVID-19 impacted major spending categories, where spending increased for additional resources in personal protective equipment (PPE), intensive care unit ventilators and intensive care unit (ICU) beds [13]. This led to significant monetary costs, where national health spending overshot government estimates by 0.8% [13]. COVID-19 led to limitations in delivering hospital services and increased costs of delivery. The annual percentage of funding towards hospitals out of the healthcare expenditure has been steadily decreasing, suggesting a shift in prioritization in healthcare spending allocations. As shown in Figure 3, percentage allocation towards hospitals have seen observable changes, decreasing more than [percent] across provinces.

**Figure 4:**
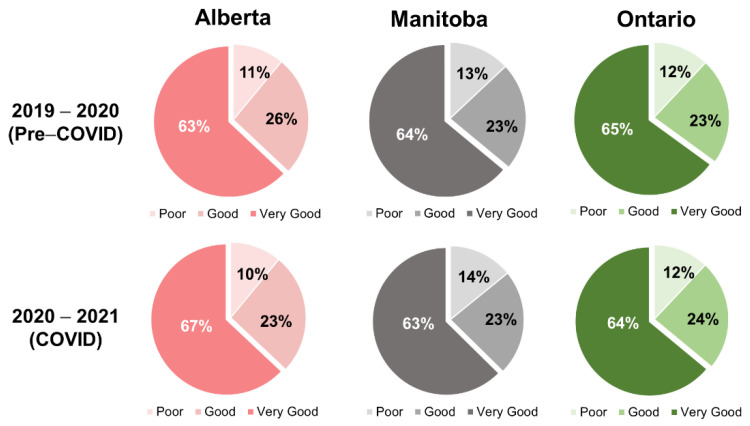
Patient survey rating hospital service experiences through 2019–2021.

In addition, we examined the patient experience data from provincial government (Figure 4). Surprisingly, patient experience remained largely unchanged, or even improved slightly, throughout the period of the pandemic in Canada. In all the provinces with available patient experience data, the percentage of survey respondents who indicated their hospital experience was “Very Good” remained relatively unchanged throughout the pandemic, with the largest satisfaction decrease at only 2% in Manitoba. The communication experience has decreased by 1%, with 55% of patients reporting that they themselves were involved as much as they wanted to be in decisions about their care [17].

## 4. Discussion

COVID-19 had tremendous impacts on the funding and delivery of healthcare services. From our results, we found that hospital occupancy was significantly reduced in reaction to COVID-19 cases. With the reduced occupancy leading to delayed and cancelled services, we expect the patient service experience to be negatively implicated. However, survey results demonstrate that patients’ own assessments of their hospital experiences remained relatively unchanged and positive.

This finding is different from some studies conducted in the United States, which found a negative correlation between patient satisfaction and wait time [18,19,20]. Logically, it is straightforward to explain why patient experience remained at a consistent level while there were limited hospital services and funding priorities shifted away from hospitals during the pandemic. One possible explanation is the implementation of new healthcare strategies to alleviate the implications of COVID-19 constraints, for example, digitalized healthcare system and technologies, which effectively replaced in-person hospital visits for disease diagnosis, monitoring and management.

### Digital Health Implementation in Canadian Hospitals

Canada has started to implement digital health technologies since 1998 [21]. Digital health technologies, including mHealth, eHealth, and wearable devices, are used to improve people’s health and provide basic health services [22]. In Canada, the main model of virtual care is virtual walk-in clinics [23]. In virtual walk-in clinics, patients log into a provider app and are matched with a licensed physician with which did not have a pre-existing clinical relationship. In British Columbia, Alberta and Ontario, virtual care is largely offered in Babylon by Telus Health, whereas Saskatchewan uses Lumeca consultations. Some provinces insured free virtual visits and some required patient payments. A popular platform, Maple, charges patients USD 49 for weekday visits and USD 79 for weekend visits. Prior to COVID-19, Canadians expressed the desire for expanded virtual options; the Canada Health Infoway Survey found that 41% of Canadians wanted virtual visits with their provider, but only 4% of family doctors offered virtual visits as an option [24]. 

As a result of the decreased hospital capacity, virtual care became a key tool for primary care physicians and specialists to triage patients virtually. Physicians accelerated the adoption of virtual care, launching online diagnosis, outpatient appointments, and other services that allowed for physicians to connect with patients remotely. Between 27% and 57% of physician services were provided virtually during the pandemic across the five provinces where data are available [2]. Within patient-reported visits between January 2021 and March 2022, 33% were virtual and 90% of patients who experienced virtual care were satisfied with their experience [25]. In this group, 21% of virtual visits were delivered through video conferencing apps. A total of 49% of Canadians reported they were offered virtual care visits along with other non-virtual modalities, and 24% reported that they were offered virtual care only [26].

Surveys on patient-clinician engagement further corroborate the effectiveness of remote diagnosis, finding equal engagement in virtual visits compared to in-person visits [27]. National survey data reflect similar results, showing an increased preference for virtual visits since the beginning of the pandemic, with almost 40% choosing virtual contact options as the ideal initial consultation method post-pandemic [28]. McKinsey’s patient-physician survey indicates that 55% of patients agree with the statement that they are much more satisfied with telehealth care versus in-person care [29]. Virtual care played a role in the maintained communication experience, increasing patient satisfaction with hospital experiences. 

The hypothesis that digital healthcare has served as an effective replacement for in-person visits is also supported by telehealth service providers data. The Center for Metabolic and Bariatric Surgery (CMBS), the sole bariatric surgery program in Manitoba, Canada, transitioned all appointments to being virtually offered. Using patient-reported experience measure (PREMs), patient satisfaction was concluded to be 81.7% [30]. Additionally, Doctors on Demand, Inc., a popular telehealth company, saw considerable increases in total visit volume associated with pandemic waves [31]. When the option of in-person visits was limited, digital health technologies became essential as a substitute visit method with high patient satisfaction.

## 5. Recommendation

Various results suggest that digital healthcare technology improves patient experience and enhances the delivery of hospital services. Its significance was highlighted in the pandemic, allowing for diagnosis without risk of transmission and risk of exposure for patients and physicians. Moreover, beyond its pandemic-contextualized benefits, digital health is cost-effective in and provides clear benefits in hospital systems.

Digital healthcare reduces the cost of healthcare to patients and improves healthcare coverage. High treatment costs and geographical distance are major barriers to healthcare access [32]. By funding digital health systems, hindrances to healthcare coverage are alleviated through increased affordability and availability. Remote areas with limited medical care facilities, remote consultation and diagnosis can access medical services with digital healthcare. This improves healthcare equity, as people in rural areas, who tend to be older and have lower socioeconomic status and education levels, can access healthcare more easily [33]. Digital health also reduces the costs of hospitals, as administration costs and transaction costs are lowered with the provision of virtual healthcare services. With virtual payment collections and the mobile application of health insurance contributions [34], the overall transaction cost of offering services can be reduced. To facilitate increased access, consideration should be given to reallocating expenditure from traditional administrative costs to digital healthcare platform costs.

Beyond cost reductions, digital health improves the overall efficiency of healthcare services. By eliminating the geographical barrier of healthcare access, patients can access a wider variety of providers, who will better match their medical needs [32]. This allows for better physician–patient matching that allows for patients to be treated by physicians in that area of expertise and maximizes the efficiency in treating a health concern. Digital health is cost-efficient and contributes to positive patient experiences in hospitals.

Post-pandemic, hospitals are resuming to pre-pandemic levels of capacity and offering of services. However, due to current inflationary pressures and limited funding allocations, hospital services have significant costs. By continuing the adoption and development of digital health tools, hospitals can improve their operations’ cost-effectiveness and enhance patient experience. With this in mind, we advocate for the broad use of digital healthcare tools in information management, service accessibility and service quality. In the discussion below, we analyzed these key pathways from regulatory, financial, and technological perspectives to advance the benefits brought by digital health integrations.

### 5.1. Information Management System

Patient information management is a critical aspect in supporting the wide-scale use of digital healthcare. With more prevalence of digital health technologies, clear regulations must be set for uniform regulation. Currently, digital health is offered by hospitals individually, resulting in a variety of delivery formats through regulated and unregulated platforms. To ensure a secure system for wide-scale usage, guidelines and legislations should be established in accordance with privacy acts such as the Personal Information Protection and Electronic Documents Act (PIPEDA) and Personal Health Information Protection Act (PHIPA).

In addition, support should be provided to simplify physician registration and licensure processes by regulatory bodies. The enhancement of physician information systems will further increase efficiency the of virtual care. Given physicians’ contrasted preferences in offering virtual care [29], physician registration should factor in comfort when offering telehealth services. The preferences of physicians should be accounted for in account registration, and more control should be given in deciding how much virtual care to offer. Through this format, there would be more engagement from physicians offering virtual care and delivering a more engaging session with patients.

Blockchain technology can be incorporated in the information management recommendations. Due to the non-tamper ability characteristic of blockchain technology [35], its further use in electronic medical record management and health data analysis would improve the efficiency of remote-diagnosis management. PwC estimates that blockchain will benefit the healthcare sector by USD 574 bn by 2030 [36]. By capitalising on the efficiencies of blockchain, transaction costs can be significantly reduced and increase the efficiency of patient system management.

By bettering the fundamental identity management aspect of digital healthcare, the safety and quality of virtual care can be improved. Moreover, these recommendations improve the national standards for patient health information access and broadly enhance identity management.

### 5.2. Accessibility of Service

A critical issue with the current digital health system is the lack of equitable access; a demographic analysis of popular digital health platforms reveals the majority of users to be younger, healthier, and living in wealthier areas [29,37]. Fees associated with remote diagnosis are a major barrier to digital healthcare access [32,34]. To improve accessibility, proper reimbursements should be given to healthcare providers, who can offer their services at more affordable levels. Provincial and territorial governments should collaborate with key associations to develop fee standards for revenue neutral services. Moreover, the registration and facilitation of digitalized civil systems in digital healthcare allows for a more comprehensive information management system. As a result, the process of identifying and targeting eligible beneficiaries for health coverage programs can be more easily facilitated. This could identify beneficiaries and form a starting point for providing equitable access to individual patients.

As outlined, increased digital health funding should be specifically targeted towards equitable access to digital healthcare. On a general level, digital health financing should implement widely agreed-on health-financing principles for funding expansion [38], including public financing, expanding prepaid and pooled funding. Such financing aligns closely with the health financing principles recommended by the World Health Organization. In addition, the Canadian government should collect data to analyze equity in accessing digital healthcare, given the current lack of aggregated data to conclude the national status quo [37]. After the collection of aggregated statistics, we recommend that further research is conducted in the analysis of accessibility improvement mechanisms specifically targeted towards the status quo.

### 5.3. Quality of Service

The information system and accessibility improvements will contribute to a better quality of service by standardizing the information and delivery of digital healthcare. In addition to these recommendations, service adaptations and technological integrations for virtual care will further improve patient experience. Beyond suggestions for clinicians’ service adaptations to integrate virtual care characteristics, we suggest that technological integrations virtually support patient experience. Healthcare providers face a challenge in supporting patients’ diagnosis needs in due to the difference in the meeting format. For example, in a physical diagnosis, the physician can ask the patient to reveal their injuries and lean closer to take a clearer examination. In a remote setting, would patients feel as comfortable undressing to show their injuries, and how would physicians adapt to a virtual examination? In a physical waiting room, patients could sit and wait until the physician called them. In a remote setting, who would give cues to these patients if another appointment is running late? Although the details could be highly specific, these are processes that have gained familiarity across physical healthcare settings. Virtual services require standardization so that patients can instill familiarity and trust in the system.

In addressing such challenges, not only do physicians need to train and adapt their services, but technological integrations could also increase the efficiency of delivery. Due to the virtual setting, technological integrations could be used as effective substitutes for traditional walk-in methods. For example, AI-based chatbots are an efficient integration that are already being used by some digital healthcare systems, where physician signals would prompt chatbots to communicate with the patients. This would help manage patient tracking and system integration, improving the overall patient experience. Moreover, technological integrations extend to an adaptation of virtual devices to conduct virtual services. Wearable devices, a model under digital health, could aid in the continuation of remote health-monitoring. Remote patient-monitoring devices, such as smart watch, RPM, and tele-homecare, enable health providers to monitor patient conditions outside of physical clinical settings and could remotely extend related diagnoses. Such integrations provide alternatives to patients, who may feel more comfortable in virtual rather than physical care. Physicians can engage more efficiently and more deeply with patients because of these tools and improve the overall effectiveness of virtual care. Given that patient engagement is critical to improving the quality of healthcare [39], we suggest that technological integration is a critical element in enhancing the patient experience in virtual care.

## 6. Conclusions

We analyzed various aspects of the Canadian healthcare system to realize significant funding allocation challenges in the pandemic period. Through compiling aggregate data on COVID-19 waves, hospitalization, patient experiences and hospital findings, we found that patients remained satisfied, despite setbacks in hospital services. We find that the results of the study support the claim that digital health effectively reduces costs and increases patient satisfaction when funding and service delivery are limited. We believe that the maintenance of the healthcare standard is, in part, a result of the digital health implantation in Canada. Our in-depth analysis and discussion further suggest three fundamental pathways to improve the effectiveness of digital healthcare. Our study offers insights to regulatory agencies regarding strategic decision-making, as well as hospitals, to improve the overall efficiency of services and reduce healthcare costs.

## Data Availability

Not applicable.
